# Empowering Students against Ethnic Bullying: Review and Recommendations of Innovative School Programs

**DOI:** 10.3390/children10101632

**Published:** 2023-09-30

**Authors:** Qiyue Wu, Fanli Jia

**Affiliations:** 1 School of Health in Social Science, The University of Edinburgh, Old College, South Bridge, Edinburgh EH8 9YL, UK; q.wu-48@sms.ed.ac.uk; 2 Department of Psychology, Seton Hall University, 400 S Orange Ave, South Orange, NJ 07079, USA

**Keywords:** ethnic bullying, intercultural contact, program, intervention, school, diversity

## Abstract

Despite research on anti-bullying interventions, there is no systemic approach or resources for teachers to address ethnic and race-related bullying in schools. In this article, we selectively reviewed theories and programs to help teachers identify and address ethnic bullying in their classrooms. We provide recommendations for workshops (e.g., cultural awareness training, empathy-building activities, bystander intervention, and stigma-based intervention). These anti-ethnic bullying workshops should promote understanding of different cultures, strengthen empathy for those who are different, encourage bystanders to take action, and reduce stigma and stereotypes. Through the sharing of diverse perspectives, expertise, and experiences, we hope this article can cultivate interactive dialogues and collaborations between educators and researchers to effectively address ethnic and race-related bullying.

## 1. Introduction

Globalization has facilitated the more frequent movement of people between countries in the present day, enhancing the process of migration. Approximately 1 out of 30 people in a global population of 7.7 billion were international migrants in 2019. Furthermore, a growing number of children and teenagers are members of ethnic minorities attending school today [1]. Globalization and migration processes have created a more diverse and multicultural society, with an ever-increasing number of children and adolescents from ethnic minority backgrounds attending schools around the world [1]. Our communities have benefited from this diversity, but it has also brought about new challenges, such as ethnic bullying [2,3]. Students who are members of racial or ethnic minorities may be bullied, victimized, and excluded by their peers [4].

As cultural and ethnic diversity increases in school settings, ethnic bullying is a growing concern [5,6]. Ethnic bullying is a type of aggression directed at individuals based on their ethnic origins [7]. In turn, this can negatively impact a student’s mental health and academic performance, effects which may last into adulthood [8,9]. As global migration continues to increase and school environments become more diverse, the urgency of addressing this issue has been highlighted [3].

Positive school environments have been shown to reduce bullying and victimization across diverse groups through a greater disciplinary structure, high academic standards, and teacher support [10]. In addition to respecting differences among students, exposure to racial and ethnic diversity is associated with a reduction in bullying reports [11,12]. Creating a respectful culture and maintaining a diverse student population can help prevent bullying in schools. The negative opinions and expectations about ethnic minority students in schools may negatively impact bullying and school culture. The prevalence of ethnic bullying decreased in schools with a positive climate and more diverse teachers [13,14].

Although researchers have developed anti-bullying interventions, there is no specific strategy in place to address ethnic and race-related bullying at the school level [15,16]. In addition, many anti-bullying programs were not culturally appropriate nor accessible to schools with a diverse student population [17]. An anti-ethnic bullying program implemented by schools should clearly outline steps to provide teachers with the tools and resources necessary to identify and prevent ethnic bullying. In this article, we discuss both classic theories and recent studies on traditional and ethnic bullying. By combining theories and practices, we can create a comprehensive and dynamic framework for understanding and combating ethnic bullying. In the following sections, we selectively review the literature of ethnic bullying and recommended four anti-ethnic bullying programs, including (1) cultural awareness training, (2) empathy-building activities, (3) bystander intervention workshops, and (4) stigma-based interventions. We intend to cultivate interactive dialogues and partnerships between educators and researchers through the discussion of diverse outlooks, expertise, and experiences.

## 2. The Risk of Ethnic Bullying in Schools

Ethnic bullying, a form of bias-based bullying, targets an individual’s race, ethnicity, or cultural background, which can result in more serious and lasting consequences than non-biased bullying [18]. A variety of detrimental physical, emotional, and mental outcomes have been linked to this type of bullying [19,20], including depression, suicidal ideation, self-harm, and substance abuse. Ethnic bullying can also negatively impact a victim’s academic performance and school-related problems, illustrating the importance of addressing this issue in educational settings [21,22,23].

The most common example of bias-based peer victimization is racial harassment and bullying [15,24,25]. When compared with general bullying, ethnic, racial, or bias-based bullying has a greater impact on mental health, harmful behaviors, and adjustment issues. According to Carter’s theory of race-based traumatic stress [15,19,20,26], racial discrimination may cause emotional, psychological, and even physical harm to its targets. Bullying can have negative health effects when combined with additional stressors, such as race-based trauma [15,16]. It has been observed that bullying at school is associated with poorer mental health outcomes in Canadian adolescents, particularly among immigrant children (as compared with non-immigrant children) [27]. Additionally, white students were less likely to drop out of school as a result of peer victimization, compared with African American and Latino students, who were more likely to do so [28]. It has also been shown that Chinese and other ethnic minority adolescents experiencing bias-based bullying were more likely to experience depression, suicidal ideation, and injury because of victimization [29]. In U.S. schools, Black and Latino students experience poor self-esteem, self-harm, illegal drug use, and depression [15,24,30,31].

In addition to adversely affecting health outcomes, ethnic bullying may also negatively affect academic and school performance. Several studies have highlighted the negative consequences experienced by ethnic minority students in relation to their educational experiences and outcomes. In a study conducted by D’hondt and colleagues [21] in Belgium, ethnic minority students who faced ethnic harassment were more likely to feel a diminished sense of belonging at school. This feeling of exclusion and alienation can have a significant impact on their academic engagement, motivation, and overall school performance. In addition, longitudinal studies conducted in Sweden have demonstrated that prolonged exposure to ethnic bullying can result in a decrease in self-esteem and lowered academic expectations among immigrant students [32]. Experiencing discrimination and victimization on a regular basis can undermine their confidence, diminish their aspirations, and hinder their progress in the classroom.

Furthermore, the cultural context and knowledge of students plays a significant role in their experience of ethnic bullying. Research conducted by Rivas-Drake and colleagues [33] found that Latino students who had a strong connection to either Latino or American culture reported more positive experiences and a reduced perception of prejudice within their schools. On the other hand, students with limited cultural knowledge or identification with either culture were more likely to perceive high levels of prejudice and to have fewer positive experiences within the school environment. It is likely that these negative experiences will further exacerbate the impact of ethnic bullying on their academic performance and overall school experience.

## 3. Developing Anti-Ethnic Bullying Intervention Programs

Schools have implemented anti-bullying programs to combat the harm caused by bullying [34]. Traditional bullying was prevented and reduced by roughly 19–20% in most of these anti-bullying programs. Approximately 15–16 percent of victimization was reduced by the programs [35]. For example, the Olweus Bullying Prevention Program (OBPP) is a comprehensive, school-based intervention program that reduces bullying in schools, prevents the recurrence of bullying problems, and improves peer relations [32,36,37]. The school environment has been restructured in order to achieve these goals. As described by Olweus [36,37], the purpose of this restructuring is to reduce the opportunities and rewards for bullying within the school environment while fostering a sense of community. Four major principles underlie the OBPP. When teaching students at school (and at home), adults should (a) maintain a warm and positive relationship; (b) set clear limits on unacceptably aggressive behavior; (c) enforce rules in a consistent, nonphysical manner; (d) provide positive role models and authority figures [36,37,38]. Four levels of intervention have been developed based on these principles: school, classroom, individual, and community, across different cultural contexts [38].

While these programs may be attributed to the relatively small number of ethnic minority students in comparison to their native counterparts, the effectiveness of these anti-bullying programs does not extend to reducing victimization among students from ethnic minority communities [15,16,17,21,39,40]. In the following section, we provide suggestions and resources regarding theoretical, methodological, and practical aspects of anti-ethnic bullying program design. These suggestions are not comprehensive, and alternative tools can be considered based on the requirements of the specific programs and cultures in schools. A multidisciplinary approach involving mental health professionals, teachers, and families will be crucial to the success of the intervention.

## 4. Theoretical Orientation of Anti-Ethnic Bullying Programs

The theoretical framework for designing effective ethnic bullying prevention programs should take into account social, cognitive, and environmental influences on student behavior. We selected several well-established theories and models, including Social Learning Theory, Contact Theory, Stigma Theory, and the Proactive Bystander Intervention Model. Based on these theories, it is possible to understand the complex mechanisms underlying ethnic bullying, in addition to devising strategies for addressing and preventing such acts.

According to the Social Learning Theory [41], individuals learn and model behaviors by observing others, particularly those they perceive as influential or similar to them. Family members could serve as role models in anti-bullying programs. In an intervention program titled “Working with Parents in Creating a Please school”, van Niejenhuis et al. [42] designed a training course with a toolkit for teachers to cooperate with parents to reduce bullying behaviors. Although the program did not yield a change to teachers’ competence or students’ victimization, parent-school cooperation had a positive effect on parents’ perception and communication with their child regarding bullying. Similarly, Gomez et al. [43] invited family members with minority backgrounds (Romanian and Arab Muslim adults) as role models to schools and worked with children (e.g., participating in learning activities, sharing cultural stories, et cetera). They found that participation in these activities as role models helped reduce cultural stereotypes and bullying at school.

Contact Theory [44] proposes that increased interaction and positive contact between individuals from diverse backgrounds can reduce prejudice and improve intergroup relations. Intergroup contact, cooperation, and communication among students of various ethnic backgrounds is incorporated as part of the intervention design, fostering a more inclusive school environment. For example, Ruck et al. [45] and Killen et al. [46] found that intergroup contact raised the awareness of stereotypes and reduced interracial peer exclusion (e.g., lunch, party, dance). Killen et al. [46,47,48] argued that schools should design programs to promote intergroup friendships and encourage students to reflect on interracial peer experiences, which in turn promotes mutual respect. In their Developing Inclusive Youth program across 48 classrooms and six schools, they used the interactive web-based course for intergroup contacts across races and ethnicities (e.g., excluding a peer who is an immigrant at bowling games). Following each video, several prompts such as feeling, judgment, decision, and reasoning were asked. They found that the intervention program was effective at changing children’s attitudes and recognizing the wrongfulness of interracial peer exclusion.

According to Tajfel et al. [49], stigmatized attitudes and behaviors are shaped by social norms, stereotypes, and individual beliefs. Intervention design should include stigma-based interventions that aim to reduce ethnic bullying by addressing social norms, stereotypes, and prejudice. Through activities that challenge biases and promote inclusivity, the intervention aims to modify the cognitive and social factors that contribute to ethnic bullying. For example, Earnshaw et al. [17] conducted a systematic review of stigma-based bullying intervention. They found 21 intervention programs (between 2000–2015) that were associated stigma-based bullying (e.g., disability, sexual minority, and physical appearance), but only one program focused on racial/ethnic bullying.

A bystander intervention model [50] emphasizes the importance of bystanders’ involvement in the prevention and resolution of ethnic bullying. During the intervention design, students are exposed to activities designed to enhance their understanding of their roles as bystanders and provide them with the necessary tools to intervene effectively in cases of racial discrimination or ethnic bullying. For example, Moran et al. [51] conducted a brief bystander intervention for anti-ethnic bullying toward Hispanic students. The program included using culturally relevant language, role-playing bullying experiences, and training in diverse values and norms. The researchers found that students participating in the anti-bullying intervention reported an increase of knowledge and confidence to intervene in ethnic bullying. Priest et al. [52] designed a “Speak Out against Racism” program to promote effective bystander responses to racial bullying in schools. The program included school policies, community involvement, curriculum designs, and training for teachers, parents, and students regarding knowledge and practical skills to reduce racism at school. They found the program effectively increased the proactive bystander responses to intervening in racial bullying.

Through the integration of these theoretical perspectives, the intervention design should attempt to address and prevent ethnic bullying in schools in a comprehensive manner. By promoting positive intergroup relationships, challenging stereotypes, encouraging inclusion, and empowering students to become proactive bystanders, the potential program should promote positive intergroup relationships. By combining these elements, the goal of any intervention programs should aim to create an academic and social environment that is supportive of students of all backgrounds and free from the damaging effects of ethnic bullying.

## 5. Recommendations of Anti-Ethnic Bullying Programs

The tasks and programs for anti-ethnic bullying were generated through a selective review above. Our aim was to make recommendations based on both traditional anti-bullying programs and recent research on racial bullying. There is a common interest among these programs in providing students with the necessary knowledge, skills, and strategies that will enable them to effectively deal with bullying situations. Additionally, these programs emphasize the need to create a safe and supportive learning environment and a culture of respect and acceptance of diversity for all students. By combining these two areas of expertise, we can formulate an effective strategy for dealing with and combating anti-ethnic bullying.

### 5.1. Cultural Awareness Workshops

Different cultures, customs, and traditions should be introduced to students at school through workshops that promote appreciation and respect for diversity. Understanding various cultures and the unique characteristics of each should be expected of students.

This workshop includes five parts: group cultural presentations, individual storytelling, cultural trivia, cultural food tasting, and a world map activity. Using a combination of experiential learning [53] and cooperative learning [54], this workshop has been developed based on a concept known as social identity theory [55], which posits that individuals derive a sense of belonging from belonging to a social group. During the workshop, students participate in hands-on activities, such as food tastings and cultural presentations, which allow them to actively engage with and learn about diverse cultures [53]. Students benefit from activities such as group presentations and cultural trivia, which promote teamwork, interdependence, and shared responsibility among them [54]. It aims to establish an inclusive and diverse environment where students from minority cultures feel accepted and welcomed, thereby reinforcing a positive social identity and reducing ethnic bullying [56].

Students can be divided into groups for a presentation on a specific culture. Next, students will share stories, folktales, or personal experiences reflecting their culture. They will be encouraged to ask questions and listen actively in order to learn about the speaker’s culture. Afterward, students will answer questions about the speaker’s culture in a trivia game. This activity promotes friendly competition and teamwork in addition to teaching new information. A cultural food tasting will follow, where students can sample and learn about traditional dishes. Each dish will be discussed in relation to its ingredients, preparation methods, and cultural significance. At the end of the lesson, students will be provided with a world map and asked to locate and label countries from which classmates, school personnel, or members of the community originate. Students from minority cultures can feel peer support, become aware that their ethnic background is accepted and welcomed, reduce their negative feelings, and establish a sense of belonging. Additionally, students can develop a newfound understanding and respect for different cultures, which can help prevent and decrease ethnic bullying in schools.

### 5.2. Empathy and Perspective Building Activities

A study by McLoughlin and Over [57] demonstrated that encouraging children to mentalize about perceived outgroup results in increased prosocial behavior towards outgroup members. This study suggests that when children were asked about the thoughts and feelings of members of immigrant groups or to explain their actions, there was an increase in their willingness to share with a novel member of the immigrant group who had been the victim of a minor transgression. This finding led to empathy-building activities. The section can be held on a weekly basis, divided into multiple sessions. Each weekly event begins with a session in identifying different feelings and how others may feel. Students will be divided into pairs or small groups. Each group will be given a list of emotions (such as happiness, sadness, anger, fear, embarrassment, etc.), and instructed to take turns acting out an emotion while their group members attempt to guess what emotion they are portraying. A component of this activity will assist students in developing their emotional literacy. They will become more aware of other people’s feelings when they recognize and understand different emotions [58].

The next part of the activity is a perspective-taking task. Students will be divided into small groups by their teachers, and each group will be assigned a scenario relating to ethnic bullying. Teachers will instruct groups to discuss the scenario from the perspective of the target, perpetrator, and bystander. Following the discussion, each group will present their findings to the entire class. The purpose of this activity is to encourage students to consider multiple perspectives and foster empathy for those who have been victimized by ethnic bullying. Students are encouraged to consider multiple perspectives in scenarios related to ethnic bullying, fostering empathy toward those involved [59,60].

The next activity will be the empathy mapping activity. Each group will be provided with poster paper and markers by the teachers. The teacher will instruct students to create an empathy map for a person experiencing ethnic bullying, addressing the following questions: (i) What do they think? (ii) What do they feel? (iii) What do they say? (iv) What do they do? Teachers will encourage groups to think about the emotional, physical, and social consequences of ethnic bullying. Students will be asked to present their empathy maps to the whole class after they have completed the empathy maps. Through this activity, students will gain a better understanding of the impact of ethnic bullying on individuals, as well as foster empathy. Empathy maps can help students understand the emotional, physical, and social consequences of ethnic bullying, fostering empathy for those who are affected. This activity was developed based on the concept and techniques of empathy mapping, which helps teams develop a deep, shared understanding and empathy for others [61].

Finally, there will be a reflection and action planning session [62]. Each teacher will distribute sticky notes to students and instruct them to write one thing they learned from the workshop, as well as one action they will take in order to promote empathy and prevent ethnic bullying in their school. All students will be invited to share their reflections and action plans with the class. As a reminder of the students’ commitments, teachers will collect the sticky notes and distribute them in a prominent location.

### 5.3. Bystander Intervention Training

According to Moran et al. [51], an ethnically mixed group may benefit from a brief implementation of a bystander bullying intervention. This intervention is based on Bandura’s Social Learning Theory [41], which suggests that children imitate influential individuals’ behaviors to shape their own behavior. In their six-weekly program, researchers found a significant decrease in ethnic bullying victimization. As another example, Priest et al.’s “Speak Out Against Racism” (SOAR) initiative aims to educate primary school students about bystander responses to racism and discrimination. Students in this program learn about racism, its effects, and how to intervene when it occurs. After conducting a mixed-methods evaluation, researchers found that students gained a better understanding of racism, were able to recognize racial discrimination, and were more likely to intervene.

In line with the research, we recommend a school-wide program that focuses on proactive bystander intervention. This program will be divided into two sections: role plays and reflections. In this program, teachers will present a definition of ethnic bullying and discuss its impact on individuals and communities. Case studies and real-life examples of ethnic bullying will be encouraged by students. Teachers will encourage students to reflect on their own experiences and discuss them in pairs or small groups.

Role-plays and Scenarios: Oyekoya et al. [63] recommended that interventions reflect the perspective of the student who bullies and the student who is bullied, as well as that of the bystander, in order to demonstrate desirable intervention behaviors. Through role-playing, the students are expected to explore different perspectives and learn how to respond to bullying situations [63]. Role-playing is an effective method for teaching bystander intervention skills, as it provides individuals with the opportunity to practice intervening in a safe environment [64]. In the second part of the activity, teachers will present participants with multiple scenarios related to ethnic bullying and bystander intervention opportunities. Participants will be divided into small groups and assigned a scenario. They will analyze the situation and then participate in a role-playing exercise to demonstrate their selected strategy. Following each role-play, a group discussion will provide feedback, identify alternative strategies, and share insights.

### 5.4. Stigma-Based Intervention

A stigma-based intervention targets bullying perpetrated against individuals based on race, ethnicity, sexual orientation, or disability. The approach targets the social precursors of stigmatized behavior and shared social norms and individual beliefs that contribute to the maintenance of stigma-based attitudes [17]. However, in a systematic review, twenty-two stigma-based bullying interventions were evaluated, but only one focused on ethnic minorities [17,65,66]. For example, Aboud and colleagues [65] have demonstrated that interventions informed by intergroup contacts [44] led to positive changes in attitudes, particularly among youth from racial and ethnic minorities. McCown [66] also stated that stigma can be reduced when these conditions are met among members from different backgrounds within the group. Therefore, a stigma reduction workshop can aim to reduce ethnic bullying by interacting with peers from different racial groups.

Understanding stigma and challenging bias: As a first step, teachers will provide a brief overview of stigmas, stereotypes, prejudices, and discrimination. Ethnic bullying can be described in real-life examples with its consequences. During the discussion period, students will be encouraged to discuss how stigma and ethnic bullying harm individuals and schools alike. As a next step, students will participate in a small group activity designed to challenge stereotypes and biases related to ethnicity that may appear in schools. Based on the contact hypothesis [44,67], interactive workshops can provide students with opportunities to participate in discussions and activities that challenge stereotypical beliefs, prejudices, and discriminatory attitudes. Each group will present their findings and engage in a discussion about strategies for countering biases and stereotypes.

According to Pettigrew and Tropp [67], intergroup contact can lead to reduced prejudice when conditions such as equal status and cooperation are met. Students from different ethnicities will participate in a collaborative problem-solving activity. Teachers will divide participants into diverse groups (5–6 students per group), ensuring that each group includes students from a variety of ethnic backgrounds. In addition, each group will receive a group number for easy identification, and the problem-solving task will immediately follow. Groups will receive handouts containing problem-solving scenarios. Teachers will explain the task to the groups: they must collaboratively develop solutions to the scenarios presented in their handouts. Cultural perspectives and experiences should be incorporated into problem-solving, and teachers will instruct students to discuss scenarios, share cultural perspectives, and find a solution together. All group members will be encouraged to communicate openly, listen actively, and respect each other. Teachers will move between groups, offering support and guidance as required.

Parents & Community Involvement Campaign: Sanders and Epstein’s [68] study on the National Network of Partnership Schools demonstrated that parental and community involvement is essential for promoting positive school outcomes, including reducing stigma and ethnic bullying. The purpose of this activity is to challenge misperceptions regarding the prevalence and acceptability of certain behaviors, such as ethnic bullying, and to promote positive, inclusive behaviors [68]. Students can feel more connected and committed to inclusivity if they are involved in the creation of campaign materials. It is expected that students will reflect on their experiences and formulate their own strategies to promote diversity and inclusivity in the classroom. Positive intergroup contact, intercultural understanding, and inclusivity can be promoted in schools to reduce and prevent ethnic bullying.

## 6. Conclusions

In a multicultural society, schools play a critical role in promoting tolerance, understanding, and mutual respect. In this article, we review different frameworks that incorporate theories, programs, and practical materials to cultivate students’ empathy, compassion, and a sense of responsibility. These frameworks should enable students to proactively counter bullying while reducing discrimination and stigma. We also intend to provide schools and teachers with the resources they need to implement an effective anti-ethnic bullying program. They can prevent ethnic bullying through empathy, diversity promotion, and targeted interventions. Indeed, many schools have already established such programs. For example, Mt. Olive School District in New Jersey (where the second author resides) implemented the Equity Task Force in 2020. A wide range of topics were reported in the initiative, including creating an inclusive curriculum, reviewing disciplinary policies, fostering relationship building through social-emotional learning, and diversifying recruitments of teachers and staff [69,70]. In 2023, Dr. Sumit Bangia and her colleagues in the school district also introduced a student-led Equity & Inclusion Student Council to ensure all students have a voice and promote an inclusive learning environment. However, anti-ethnic bullying programs cannot solely rely on the participation of teachers and schools. Salmivalli et al. [71] argue that curriculum-based, class-level work is insufficient to prevent bullying behaviors in schools. Educating students about ethnic bullying requires collaboration between teachers, parents, administrators, and community members. It is possible to establish an inclusive and healthy school environment by revising curriculums to include multicultural content, providing staff training on how to handle ethnic bullying, and providing parents with guidebooks on how to promote respect and tolerance at home [15,16]. Anti-ethnic bullying can be raised by organizing programs and workshops that teach effective interventions and prevention strategies. 

In conclusion, we hope that this article will provide a selective review of ethnic/racial-related bullying in schools and serve as a starting point for further research and collaboration. We would also like to emphasize that the primary purpose of this article is not to provide an exhaustive review of the literature. As an alternative, we advocate for specific programs that address both traditional bullying and racial bullying in schools and provide educators with the necessary resources to effectively implement anti-ethnic bullying programs. Future research should examine the effectiveness of the recommended tasks in specific contexts. A systematic review or meta-analysis of existing interventions could provide additional evidence of their effectiveness. Finally, we hope that the article will serve as an informative tool for guiding policy decisions and for creating more effective strategies for preventing and addressing ethnic bullying.

## References

[1] International Organization for Migration (2019). World Migration Report 2020.

[2] Nansel T.R., Craig W., Overpeck M.D., Pilla R.S., Ruan W.J., Simons-Morton B., Scheidt P. (2001). Bullying behaviors among U.S. youth: Prevalence and association with psychosocial adjustment. JAMA.

[3] Graham S., Juvonen J. (2002). Ethnicity, peer harassment, and adjustment in middle school: An exploratory study. J. Early Adolesc..

[4] Pottie K., Dahal G., Georgiades K., Premji K., Hassan G. (2015). Do first generation immigrant adolescents face higher rates of bullying, violence and suicidal behaviours than do third generation and native born?. J. Immigr. Minor. Health.

[5] Smith P.K., López-Castro L., Robinson S., Görzig A. (2019). Consistency of gender differences in bullying in cross-cultural surveys. Aggress. Violent Behav..

[6] Tippett N., Wolke D. (2014). Socioeconomic status and bullying: A meta-analysis. Am. J. Public Health.

[7] Elamé E. (2013). Discriminatory Bullying: A New Intercultural Challenge.

[8] Moore S.E., Norman R.E., Suetani S., Thomas H.J., Sly P.D., Scott J.G. (2017). Consequences of bullying victimization in childhood and adolescence: A systematic review and meta-analysis. World J. Psychiatry.

[9] Ttofi M.M., Farrington D.P., Lösel F., Loeber R. (2011). Do the victims of school bullies tend to become depressed later in life? A systematic review and meta-analysis of longitudinal studies. J. Aggress. Confl. Peace Res..

[10] Konold T., Cornell D., Shukla K., Huang F. (2017). Racial/ethnic differences in perceptions of school climate and its association with student engagement and peer aggression. J. Youth Adolesc..

[11] Gage N.A., Prykanowski D.A., Larson A. (2014). School climate and bullying victimization: A latent class growth model analysis. Sch. Psychol. Q..

[12] Lanza H.I., Echols L., Graham S. (2018). A silver lining: The role of ethnic diversity on co-occurring trajectories of weight status and peer victimization across early adolescence. J. Adolesc. Health.

[13] Larochette A.C., Murphy A.N., Craig W.M. (2010). Racial bullying and victimization in Canadian school-aged children: Individual and school level effects. Sch. Psychol. Int..

[14] Wright M.F., Wachs S. (2019). Does social support moderate the relationship between racial discrimination and aggression among Latinx adolescents? A longitudinal study. J. Adolesc..

[15] Xu M., Macrynikola N., Waseem M., Miranda R. (2020). Racial and ethnic differences in bullying: Review and implications for intervention. Aggress. Violent Behav..

[16] Waseem M., Amanda B.N. (2023). Bullying: Issues and challenges in prevention and intervention. Curr. Psychol..

[17] Earnshaw V.A., Reisner S.L., Menino D., Poteat V.P., Bogart L.M., Barnes T.N., Schuster M.A. (2018). Stigma-based bullying interventions: A systematic review. Dev. Rev..

[18] Russell S.T., Sinclair K.O., Poteat V.P., Koenig B.W. (2012). Adolescent health and harassment based on discriminatory bias. Am. J. Public Health.

[19] Carter R.T. (2007). Racism and psychological and emotional injury: Recognizing and assessing race-based traumatic stress. Couns. Psychol..

[20] Williams D.R., Yan Y., Jackson J.S., Anderson N.B. (1997). Racial differences in physical and mental health: Socio-economic status, stress and discrimination. J. Health Psychol..

[21] D’hondt F., Van Houtte M., Stevens P.A. (2015). How does ethnic and non-ethnic victimization by peers and by teachers relate to the school belongingness of ethnic minority students in Flanders, Belgium? An explorative study. Soc. Psychol. Educ..

[22] Bayram Özdemir S., Stattin H. (2014). Why and when is ethnic harassment a risk for immigrant adolescents’ school adjustment? Understanding the processes and conditions. J. Youth Adolesc..

[23] Volk A.A., Dane A.V., Marini Z.A. (2014). What is bullying? A theoretical redefinition. Dev. Rev..

[24] Bucchianeri M.M., Eisenberg M.E., Wall M.M., Piran N., Neumark-Sztainer D. (2014). Multiple types of harassment: Associations with emotional well-being and unhealthy behaviors in adolescents. J. Adolesc. Health.

[25] de Oliveira W.A.D., Silva M.A.I., Mello F.C.M.D., Porto D.L., Yoshinaga A.C.M., Malta D.C. (2015). The causes of bullying: Results from the national survey of school health (PeNSE). Rev. Latino-Am. Enferm..

[26] Polanco-Roman L., Danies A., Anglin D.M. (2016). Racial discrimination as racebased trauma, coping strategies, and dissociative symptoms among emerging adults. Psychological Trauma: Theory, Research, Practice, and Policy..

[27] Abada T., Hou F., Ram B. (2008). The effects of harassment and victimization on self rated health and mental health among Canadian adolescents. Soc. Sci. Med..

[28] Peguero A.A. (2011). Violence, Schools, and Dropping Out: Racial and ethnic disparities in the educational consequence of student victimization. J. Interpers. Violence.

[29] Pan S.W., Spittal P.M. (2013). Health effects of perceived racial and religious bullying among urban adolescents in China: A cross-sectional national study. Glob. Public Health.

[30] Cardoso J.B., Szlyk H.S., Goldbach J., Swank P., Zvolensky M.J. (2018). General and ethnic-biased bullying among Latino students: Exploring risks of depression, suicidal ideation, and substance use. J. Immigr. Minor. Health.

[31] Rosenthal L., Earnshaw V.A., Carroll-Scott A., Henderson K.E., Peters S.M., McCaslin C., Ickovics J.R. (2015). Weight-and race-based bullying: Health associations among urban adolescents. J. Health Psychol..

[32] Olweus D. (1993). Bullying at School: What We Know and What We Can Do.

[33] Rivas-Drake D., Seaton E.K., Markstrom C., Quintana S., Syed M., Lee R.M., Schwartz S.J., Umaña-Taylor A.J., French S., Yip T. (2014). Ethnic and racial identity in adolescence: Implications for psychosocial, academic, and health outcomes. Child Dev..

[34] Farrell A.D., Sullivan T.N., Sutherland K.S., Corona R., Masho S. (2018). Evaluation of the olweus bully prevention program in an urban school system in the USA. Prev. Sci..

[35] Gaffney H., Farrington D.P., Espelage D.L., Ttofi M.M. (2019). Are cyberbullying intervention and prevention programs effective? A systematic and meta-analytical review. Aggress. Violent Behav..

[36] Olweus D., Limber S.P., Mihalic S. (1999). The Bullying Prevention Program: Blueprints for Violence Prevention.

[37] Olweus D., Limber S.P., Flerx V., Mullin N., Riese J., Snyder M. (2007). Olweus Bullying Prevention Program Schoolwide Guide.

[38] Olweus D., Limber S.P. (2010). Bullying in school: Evaluation and dissemination of the Olweus Bullying Prevention Program. Am. J. Orthopsychiatry.

[39] Bauer N.S., Lozano P., Rivara F.P. (2007). The effectiveness of the Olweus Bullying Prevention Program in public middle schools: A controlled trial. J. Adolesc. Health.

[40] Limber S.P., Olweus D., Wang W., Masiello M., Breivik K. (2018). Evaluation of the Olweus Bullying Prevention Program: A large scale study of U.S. students in grades 3–11. J. Sch. Psychol..

[41] Bandura A. (1977). Self-efficacy: Toward a unifying theory of behavioral change. Psychol. Rev..

[42] Van Niejenhuis C., Huitsing G., Veenstra R. (2020). Working with parents to counteract bullying: A randomized controlled trial of an intervention to improve parent-school cooperation. Scand. J. Psychol..

[43] Gómez A., Munte A., Sorde T. (2014). Transforming schools through minority males’ participation: Overcoming cultural stereotypes and preventing violence. J. Interpers. Violence.

[44] Allport G.W. (1954). The Mature of Prejudice.

[45] Ruck M.D., Park H., Killen M., Crystal D.S., Ruck M. (2011). Intergroup Contact and Evaluations of Race-Based Exclusion in Urban Minority Children and Adolescents. J. Youth Adolesc..

[46] Killen M., Clark Kelly M., Richardson C., Crystal D., Ruck M. (2010). European American children’s and adolescents’ evaluations of interracial exclusion. Group Process. Intergroup Relat..

[47] Killen M., Rutland A. (2022). Promoting fair and just school environments: Developing inclusive youth. Policy Insights from the Behavioral and Brain Sciences..

[48] Killen M., Luken Raz K., Graham S. (2022). Reducing prejudice through promoting cross-group friendships. Rev. Gen. Psychol..

[49] Tajfel H., Billig M.G., Bundy R.P., Flament C. (1971). Social categorization and intergroup behaviour. Eur. J. Soc. Psychol..

[50] Polanin J.R., Espelage D.L., Pigott T.D. (2012). A Meta-Analysis of School-Based Bullying Prevention Programs’ Effects on Bystander Intervention Behavior. Sch. Psychol. Rev..

[51] Moran M., Midgett A., Doumas D.M. (2020). Evaluation of a brief, bystander bullying intervention (STAC) for ethnically blended middle schools in low-income communities. Prof. Sch. Couns..

[52] Priest N., Alam O., Truong M., Sharples R., Nelson J., Dunn K., Francis K., Paradies Y., Kavanagh A. (2021). Promoting proactive bystander responses to racism and racial discrimination in primary schools: A mixed methods evaluation of the ‘Speak Out Against Racism’ program pilot. BMC Public Health.

[53] Kolb D.A. (1984). Experience as the Source of Learning and Development.

[54] Johnson D.W., Johnson R.T. (1987). Learning Together and Alone: Cooperative, Competitive, and Individualistic Learning.

[55] Tajfel H., Turner J., Austin W.G., Worchel S. (1979). An Integrative Theory of Intergroup Conflict. The Social Psychology of Intergroup Relations.

[56] Juvonen J., Nishina A., Graham S. (2006). Ethnic diversity and perceptions of safety in urban middle schools. Psychol. Sci..

[57] McLoughlin N., Over H. (2019). Encouraging children to mentalise about a perceived outgroup increases prosocial behaviour towards outgroup members. Dev. Sci..

[58] Brackett M.A., Rivers S.E., Salovey P. (2011). Emotional intelligence: Implications for personal, social, academic, and workplace success. Soc. Pers. Psychol. Compass.

[59] Ni Y., Jia F. (2023). Promoting Positive Social Interactions: Recommendation for a Post-Pandemic School-Based Intervention for Social Anxiety. Children.

[60] Galinsky A.D., Moskowitz G.B. (2000). Perspective-taking: Decreasing stereotype expression, stereotype accessibility, and in-group favoritism. J. Pers. Soc. Psychol..

[61] Gray D., Brown S., Macanufo J. (2010). Gamestorming: A Playbook for Innovators, Rulebreakers, and Changemakers.

[62] Gai X., Gu T., Wang Y., Jia F. (2022). Improving career adaptability through motivational interview among peers: An intervention of at-risk Chinese college students majoring in foreign language. J. Vocat. Behav..

[63] Oyekoya O., Urbanski J., Shynkar Y., Baksh A., Etsaghara M. (2021). Exploring first-person perspectives in designing a role-playing VR simulation for bullying prevention: A focus group study. Front. Virtual Real..

[64] Kuntz J.C., Searle F. (2023). Does bystander intervention training work? When employee intentions and organisational barriers collide. J. Interpers Violence.

[65] Aboud F.E., Tredoux C., Tropp L.R., Brown C.S., Niens U., Noor N.M. (2012). Interventions to reduce prejudice and enhance inclusion and respect for ethnic differences in early childhood: A systematic review. Dev. Rev..

[66] McKown C. (2005). Applying ecological theory to advance the science and practice of school-based prejudice reduction interventions. Educ. Psychol..

[67] Pettigrew T.F., Tropp L.R. (2006). A meta-analytic test of intergroup contact theory. J. Pers. Soc. Psychol..

[68] Sanders M.G., Epstein J.L. (2000). The national network of partnership schools: How research influences educational practice. JESPAR.

[69] Mount Olive Township School District. https://www.nj.gov/education/finance/fp/acfr/search/21/3450.pdf.

[70] MOTSD Equity Task Force. https://sites.google.com/motsd.org/motsdequitytaskforce/home?authuser=0.

[71] Salmivalli C., Voeten M., Poskiparta E. (2011). Bystanders matter: Associations between reinforcing, defending, and the frequency of bullying behavior in classrooms. J. Clin. Child Adolesc. Psychol..

